# Supplementary material for: Investigating Morphological Changes of T-lymphocytes after Exposure with Bacterial Determinants for Early Detection of Septic Conditions
Source: Microorganisms. 2022 Feb 8;10(2):391. doi: 10.3390/microorganisms10020391 (PMC8879819; doi:10.3390/microorganisms10020391)
Supplement: Supplementary file 1 [file microorganisms-10-00391-s001.zip › microorganisms-1577157-supplementary.pdf]

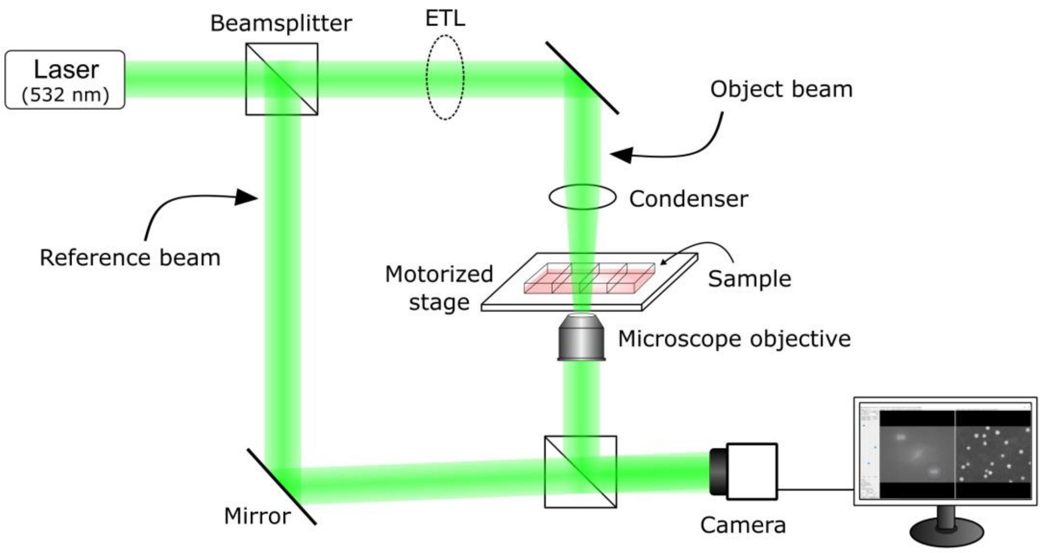

Supplemental Figure S1. DHM setup. A coherent light source (here a laser) is used to acquire digital off-axis holograms. The laser beam is divided into a reference beam that remains undisturbed and an object illumination beam that passes through the sample. The object illumination is modulated by an electrically focus tunable lens (ETL) to reduce coherence and pathogen induced image disturbances. Thereby a series of multiple holograms is recorded. Finally, the two beams are joined and the interference pattern, i.e. the hologram, is captured with a camera (a complementary metal-oxide semiconductor [CMOS] sensor).

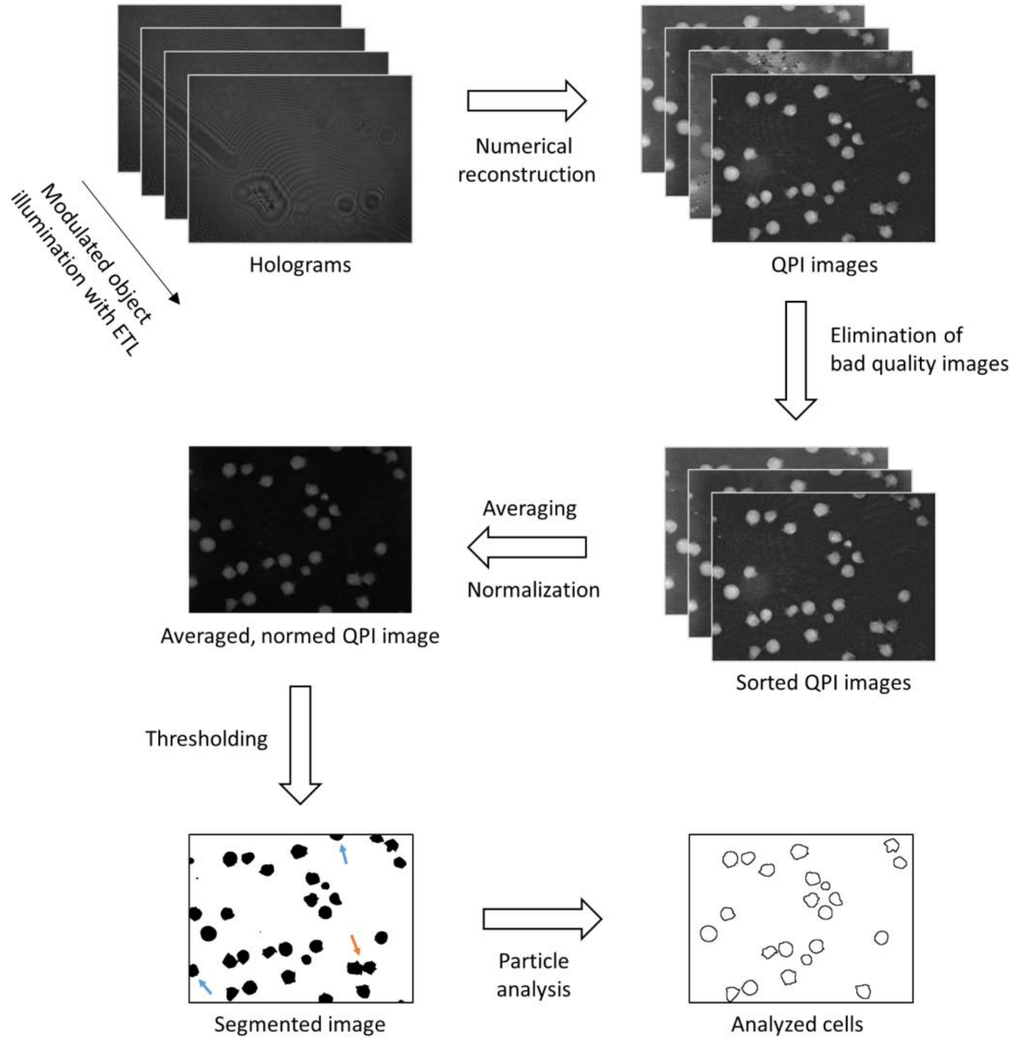

Supplemental Figure S2. Workflow of DHM data analysis. Multiple holograms were acquired while the object illumination was modulated with an electrically focus tunable lens (ETL) [47]. Quantitative phase images were numerically reconstructed as previously described [48]. After elimination of inadequately reconstructed images (sorting), averaged QPI images were calculated and normed across the time-lapse series. Subsequently, image segmentation was performed using Fiji software by adjusting the threshold to 1.1 radian. During the following particle analysis, cells touching the image borders (blue arrow) or cell aggregates (orange arrow) were excluded. Morphological parameters (e.g. area, perimeter, circularity, etc.) were measured on a single cell level based on the image segmentation. Gray values per cell (e.g. mean, min, max, etc.) were determined by redirecting the image segmentation to the original averaged and normed QPI image and transformed into phase contrast values. Results obtained from one biological replicate represent the average of about 60 single cells. QPI = quantitative phase imaging

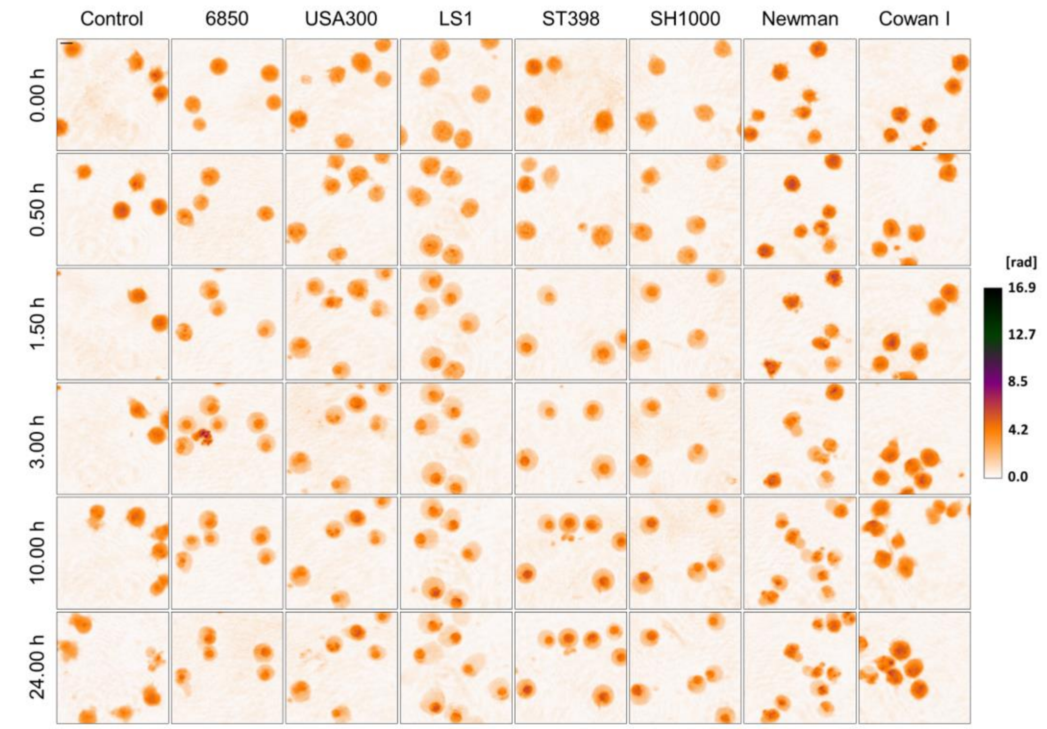

Supplemental Figure S3. Quantitative DHM phase contrast images showing morphological changes of Jurkat cells treated with MVs derived from different *S. aureus* strains. Jurkat cells were exposed to 20 µg/mL MVs of different *S. aureus* strains. Untreated cells served as control. Time-lapse DHM was applied to monitor cellular changes. Representative color-coded phase contrast images of Jurkat cells are shown at indicated time points after addition of MVs. The scale bar corresponds to 10 µm. The calibration bar indicates phase contrast values in radian.

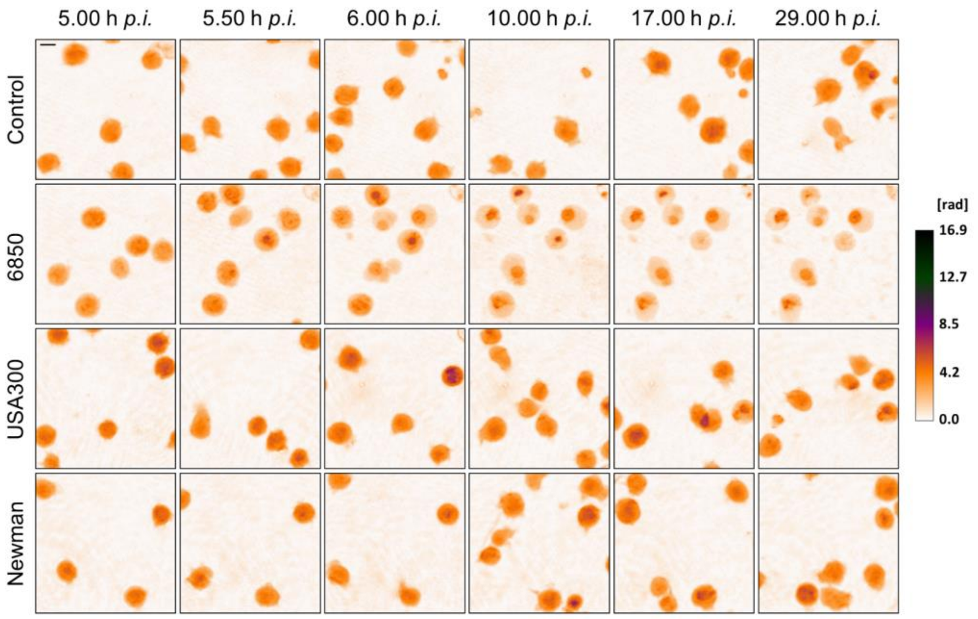

Supplemental Figure S4. Quantitative DHM phase contrast images of Jurkat cells infected with living *S. aureus*. Jurkat cells were infected with different *S. aureus* strains (MOI 10) and after 4 h, bacteria were removed by centrifugation and Lysostaphin treatment (2 µg/mL) to prevent overgrowth. Mock-infected cells served as control. At 5 h *p.i.* time-lapse measurement was started to investigate morphological changes. Representative color-coded phase contrast images display Jurkat cells at given time points after addition of bacteria. The scale bar corresponds to 10 µm. The calibration bar indicates phase contrast values in radian.

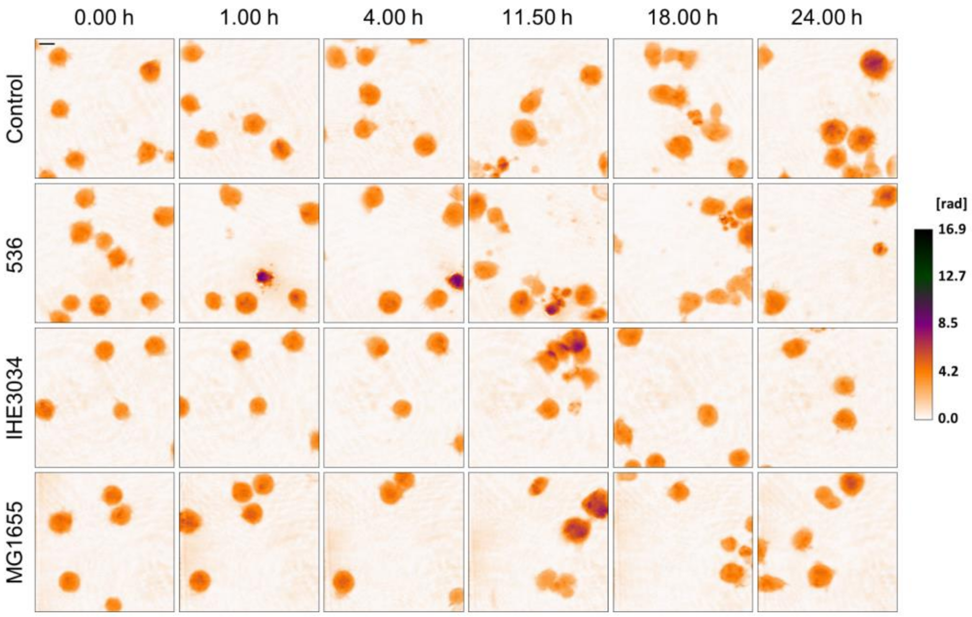

Supplemental Figure S5. Quantitative DHM phase contrast images of Jurkat cells exposed to MVs of different *E. coli* strains. Jurkat cells were treated with 20 µg/mL MVs of different *E. coli* strains while untreated cells served as control. Time-lapse investigation of cellular changes was performed using DHM. Reconstructed color-coded phase contrast images of Jurkat cells are presented at indicated time points after addition of MVs. The scale bar corresponds to 10 µm. The calibration bar indicates phase contrast values in radian.

Supplemental Table S1. Settings used for image analysis with Fiji.

Step	Fiji function	Settings
1	Background subtraction	Rolling ball radius = 60 pixels
2	Adjust threshold	Min. threshold = 1.1 radian*
3	Convert stack to binary	Method = default<br>Background = dark
4	Set measurements	Redirect to: phase contrast image
5	Analyze particles	Size = 200 – 9000 pixels<br>Circularity = 0.00 – 1.00<br>Exclude on edges

* Every pixel with a phase contrast ≥ 1.1 radian is defined as foreground and < 1.1 radian as background.
